# The identification of key genes and pathways in glioblastoma by bioinformatics analysis

**DOI:** 10.1080/23723556.2023.2246657

**Published:** 2023-08-14

**Authors:** Zahra Farsi, Najaf Allahyari Fard

**Affiliations:** a Department of Biology, Noor-Dnaesh Institute of Higher Education, Esfahan, Iran; b Department of Systems Biotechnology, National Institute of Genetic Engineering and Biotechnology (NIGEB), Tehran, Iran

**Keywords:** Brain cancer, key genes, key pathways, gene expression profiling, PPI network

## Abstract

GBM is the most common and aggressive type of brain tumor. It is classified as a grade IV tumor by the WHO, the highest grade. Prognosis is generally poor, with most patients surviving only about a year. Only 5% of patients survive longer than 5 years. Understanding the molecular mechanisms that drive GBM progression is critical for developing better diagnostic and treatment strategies. Identifying key genes involved in GBM pathogenesis is essential to fully understand the disease and develop targeted therapies. In this study two datasets, GSE108474 and GSE50161, were obtained from the Gene Expression Omnibus (GEO) to compare gene expression between GBM and normal samples. Differentially expressed genes (DEGs) were identified and analyzed. To construct a protein-protein interaction (PPI) network of the commonly up-regulated and down-regulated genes, the STRING 11.5 and Cytoscape 3.9.1 were utilized. Key genes were identified through this network analysis. The GEPIA database was used to confirm the expression levels of these key genes and their association with survival. Functional and pathway enrichment analyses on the DEGs were conducted using the Enrichr server. In total, 698 DEGs were identified, consisting of 377 up-regulated genes and 318 down-regulated genes. Within the PPI network, 11 key up-regulated genes and 13 key down-regulated genes associated with GBM were identified. NOTCH1, TOP2A, CD44, PTPRC, CDK4, HNRNPU, and PDGFRA were found to be important targets for potential drug design against GBM. Additionally, functional enrichment analysis revealed the significant impact of Epstein-Barr virus (EBV), Cell Cycle, and P53 signaling pathways on GBM.

## Introduction

Cancers arise due to inherited or spontaneous changes in genes that regulate cell processes. These genetic mutations, detectable through chromosomal analysis and cytotype analysis, often activate oncogenes or suppress tumor suppressor genes, leading to abnormal cell population growth and tumor formation.^1^

Glioblastoma (GBM) is the most common and aggressive brain tumor that originates in the supportive tissue of the brain, known as glial cells, classified as grade IV by the World Health Organization (WHO).^2^ While 95% of GBM tumors occur in the supratentorial region, only a few percent develop in the cerebellum, brain stem, or spinal cord. GBM ranks as the second leading cause of cancer-related deaths and the third leading cause of cancer deaths among individuals aged 15 to 34. The annual incidence of GBM is approximately 3–19 cases per 100,000 populations, with an average age of diagnosis being 64 years. GBM is notorious for its resistance to standard treatments, such as surgery, radiation therapy, and chemotherapy. The tumor’s ability to infiltrate healthy brain tissue makes complete surgical removal extremely challenging, and its complex molecular profile contributes to its resistance to conventional therapies. Most GBM patients survive for about one year, and only 5% survive beyond five years.^3^

Despite the challenges posed by GBM, research into understanding its underlying genetic and molecular mechanisms has provided insights that are gradually leading to more targeted and personalized treatment approaches. Additionally, innovative therapies, including targeted drug treatments and immunotherapies, are being explored in clinical trials to harness the body’s immune system to recognize and attack GBM cells specifically. Collaborative efforts between researchers, oncologists, and neurosurgeons continue to drive progress in the fight against GBM, aiming for improved outcomes and enhanced quality of life for patients facing this challenging diagnosis.

As a result, understanding the mechanisms and developing effective treatment strategies is crucial to improve the prognosis of GBM patients. Numerous investigations have been conducted to elucidate the molecular mechanisms and pathways responsible for GBM progression and growth, yet the exact processes remain elusive. Recently, genomic data analysis has gained significant attention, leading to increased understanding of GBM. Successful gene searches have revealed various biomarkers for cancer detection and early prediction, thereby offering insights into the molecular mechanisms of GBM and potential therapeutic targets. In this study, we evaluated different gene expression profiles, selected relevant profiles, and examined protein-protein interactions (PPI) to identify genes associated with GBM. Additionally, we identified key genes and genetic pathways related to GBM. Our findings may offer valuable predictive biomarkers and potential therapeutic targets.

## Material and method

**Figure 1.**
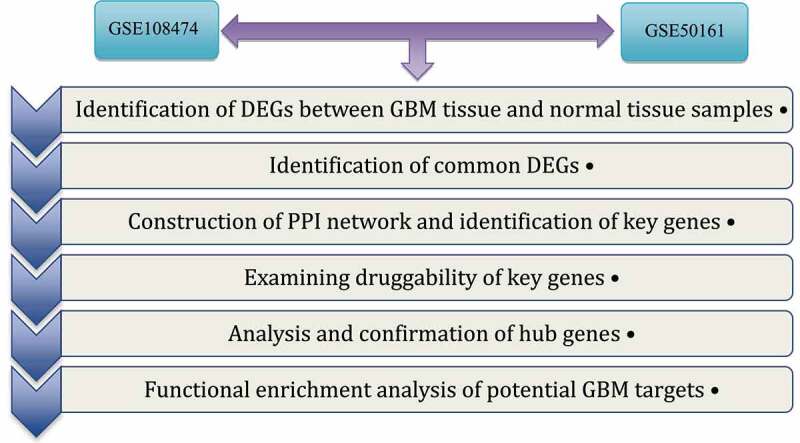
The steps to identification of DEGs and analysis of hub genes in the GBM.

In this study, we selected two microarray profiles from the GEO database with access codes GSE108474 and GSE50161.^4,5^ GSE108474 contained 219 samples, including 28 normal samples, while GSE5016134 had GBM samples and 13 normal samples. Both datasets were generated using the Affymetrix Human U133 Plus 2.0 array platform. To identify differentially expressed genes (DEGs) between GBM samples and normal samples, we utilized GEO2R (http://www.ncbi.nlm.nih.gov/geo/geo2r). Genes with significant differential expression were selected based on the criteria of adjusted P-value <.05 and logFC > 1. Subsequently, we evaluated and analyzed the overlapping DEGs shared between the two datasets using Funrich 3.1.3. To visually identify the shared genes, we plotted a Venn Diagram (Figure 1).

To create a protein-protein interaction network and identify known and predicted relationships between candidate genes, we utilized STRING 11.5 (http://string-db.org).^6^ The resulting protein network was visualized using Cytoscape 3.8.1.^7^ Subsequently, we measured the centrality of nodes within the network using the CytoHubba plugin.^8^ For the identification of drug targets, we employed the DGIdb web server. This allowed us to extract relevant information on potential drug targets associated with our candidate genes.^9^ To further validate the key genes, we utilized the GEPIA web server (http://gepia.cancer-pku.cn/). GEPIA is a powerful tool for gene expression profiling, enabling analysis of both normal and cancer genes and providing interactive comparisons between them.^10^ In addition, we used the online Enrichr server (http://maayanlab.could/Enrichr) to analyze pathways and biological processes relevant to GBM.^11^ The information was obtained from the KEGG (Kyoto Encyclopedia of Genes and Genomes) database.^12,13^

## Results

**Figure 2.**
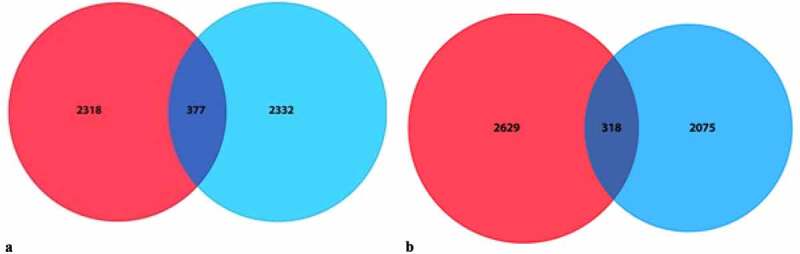
Venn Diagram showing the DEGs in two datasets. A: upregulated DEGs, B: downregulated DEGs.

**Figure 3.**
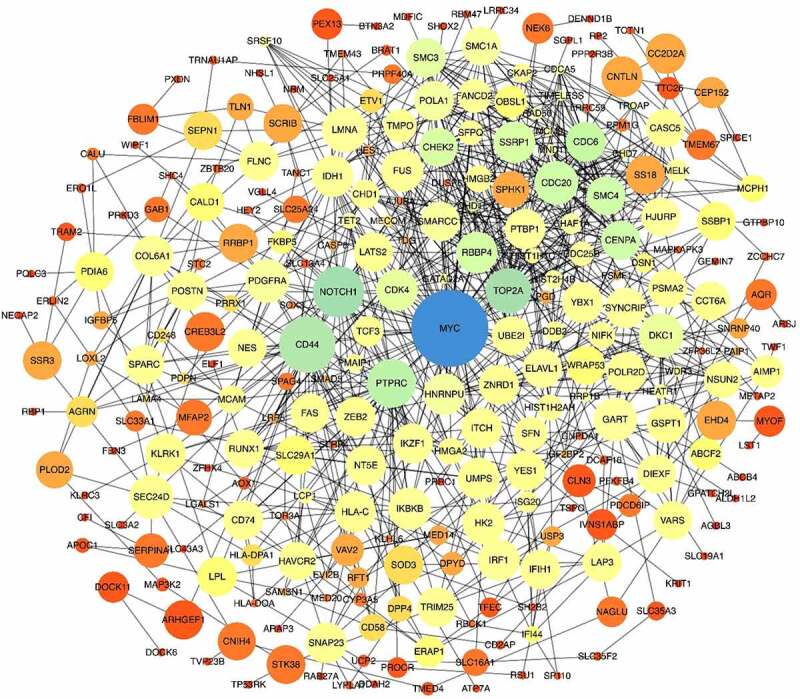
Protein-protein interaction of co-expressed DEGs. The nodes with a higher degree are shown in larger sizes and blue colors, while the nodes with a lower degree are shown in smaller sizes and orange color. The size of the edges corresponds to the strength of the interaction. MYC is the most prominent and key node in this network in terms of both indices.

A total of 25,607 genes with distinct expression levels were identified from the GSE108474 and GSE50161 profiling datasets. Among these, 3,446 genes were upregulated, and 3,802 genes were downregulated. Subsequently, we performed Venn analysis and identified 377 commonly upregulated differentially expressed genes (DEGs) and 318 commonly downregulated DEGs (Figure 2) for further analysis. Among the identified key genes, some may hold significant roles in disease prevention and development. Co-occurring DEGs were mapped using the STRING website, where the confidence score was set at 0.4. The resulting protein-protein interaction (PPI) network was further analyzed using Cytoscape (version 3.8.1). As shown in Figure 3, the PPI network consisted of 275 nodes and 914 edges (after removal of non-connected nodes). This comprehensive network offers valuable insights into the relationships between drug targets and other proteins from a systematic perspective.^14^

**Figure 4.**
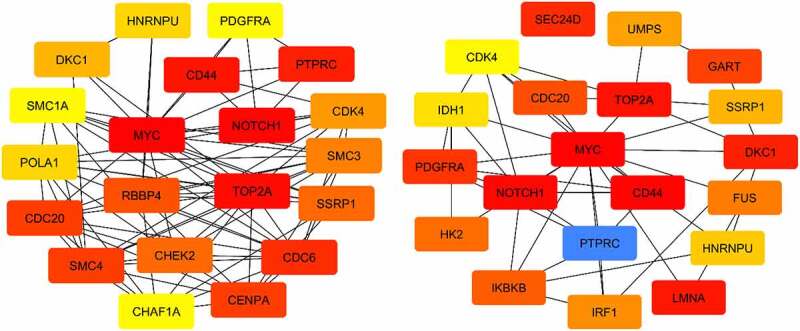
Interactions between 20 genes have been found to increase expression. Key genes were selected based on the Degree criterion (left figure) and selected genes based on the Betweenness criterion (right figure) using CytoHubba. The red color in both figures indicates a high degree and betweenness. The blue color in the right figure indicates the lowest betweenness.

**Figure 5.**
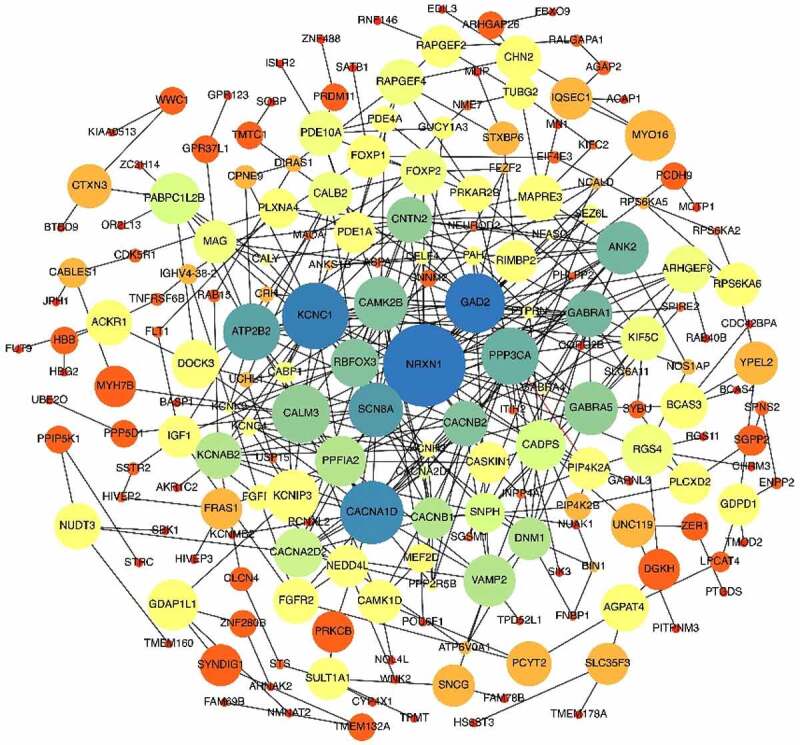
PPI network of downregulated genes obtained from the Cytoscape.

Using the CytoHubba tool, we separately filtered genes with increased expression based on the metrics of Degree and Betweenness (Figure 4). The Degree and number of edges within a network indicate the interactions of a gene with others, suggesting its pivotal position and importance. This analysis led to the identification of 20 hub genes. Also, all the steps performed to obtain the overexpressed genes were replicated to obtain the underexpressed genes (Figure 5).

**Figure 6.**
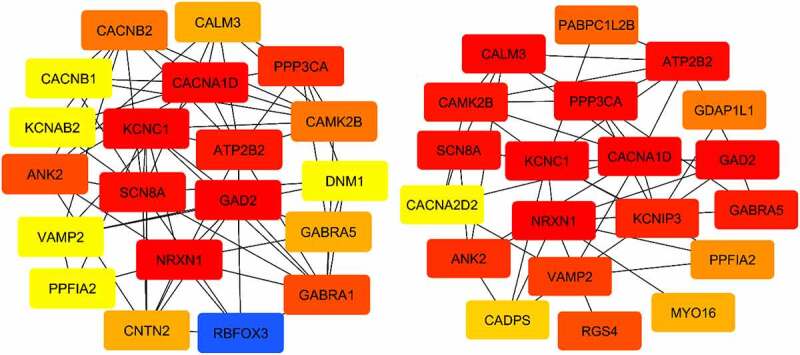
Interactions between 20 genes have been found to decrease. Key genes were selected based on the Degree criteria (left figure) and genes were selected based on the Betweenness criteria (right figure) using CytoHubba. The red color in both figures indicates a high degree and betweenness level.

Additionally, using the CytoHubba tool, genes with decreased expression, as measured by the Degree and Betweenness criteria, were identified. Figure 6 illustrates the interactions between the 20 genes with decreased expression. In this study, we employed the CytoHubba software and the Funrich program to identify genes with both increased and decreased expression. Specifically, genes with high Degree and high Betweenness were considered as key genes. We identified 11 key genes (MYC, NOCH1, TOP2A, CD44, PTPRC, CDC20, SSRP1, CDK4, DKC1, HNRNPU, PDGFRA) with increased expression, which are of significant importance in GBM. Additionally, we identified 13 key genes (NRXN1, GAD2, KCNC1, CACNA1D, SCN8A, ATP2B2, PPP3CA, ANK2, CAMK2B, CALM3, GABRA5, VAMP2, PPFIA2) with decreased expression.

Using the DGIdb web server, we examined the drug-response capability of the key genes identified in previous studies. Among the 11 key genes, 10 were identified by DGIdb, and genes such as NOTCH1, TOP2A, CD44, PTPRC, CDK4, HNRNPU, and PDGFRA were found to be potential drug-responsive or therapeutic targets (Table 1).

**Table 1.** Drug-response capability and functional classification of key proteins identified as upregulated.

Drug-response capable proteins	Protein Explanations
MYC	CLINICALLY ACTIONABLE, TRANSCRIPTION FACTOR
NOTCH1	DRUGGABLE GENOME, CLINICALLY ACTIONABLE, TRANSPORTER, CELL SURFACE
TOP2A	DRUGGABLE GENOME, CLINICALLY ACTIONABLE, ENZYME
CD44	DRUGGABLE GENOME, CELL SURFACE
PTPRC	DRUGGABLE GENOME, PROTEIN PHOSPHATASE, CLINICALLY ACTIONABLE, CELL SURFACE, TUMOR SUPPRESSOR, EXTERNAL SIDE OF PLASMA MEMBRANE, KINASE
SSRP1	DNA REPAIR, KINASE
CDK4	KINASE, DRUGGABLE GENOME, CLINICALLY ACTIONABLE, TUMOR SUPPRESSOR, TRANSCRIPTION FACTOR COMPLEX, SERINE THREONINE KINASE, DRUG RESISTANCE, ENZYME, TRANSCRIPTION FACTOR
DKC1	CLINICALLY ACTIONABLE
HNRNPU	B30_2 SPRY DOMAIN, DRUGGABLE GENOME, CELL SURFACE, TRANSCRIPTION FACTOR,
PDGFRA	DRUGGABLE GENOME, KINASE, EXTERNAL SIDE OF PLASMA MEMBRANE, CLINICALLY ACTIONABLE, TYROSINE KINASE, DRUG RESISTANCE

**Figure 7.**
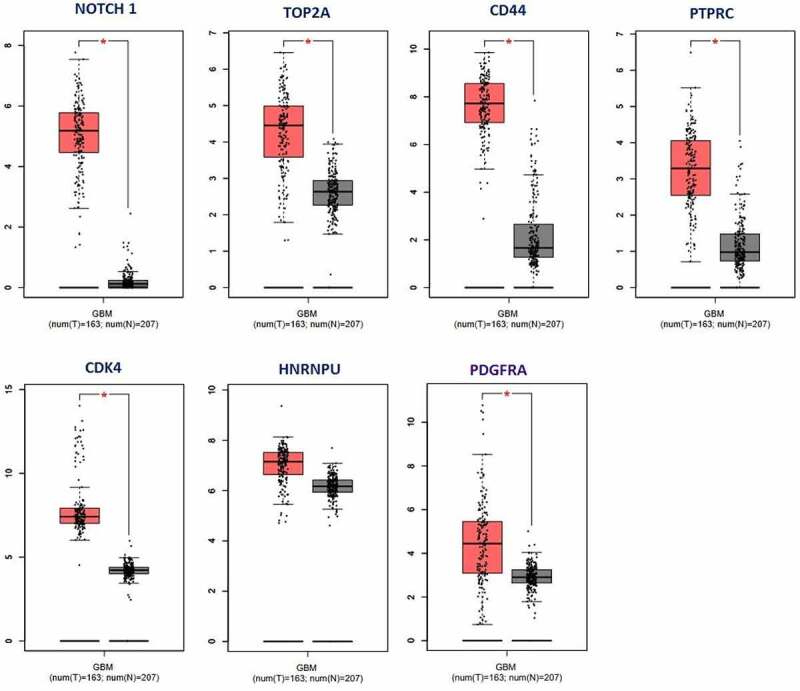
A Boxplot of the expression levels of key and druggable genes (NOTCH1, TOP2A, CD44, PTPRC, CDK4, HNRNPU, and PDGFRA) in tumor tissue (red plot) have increased compared to healthy control samples (gray plot).

To confirm the key genes involved in increased expression and druggability, the GEPIA server (http://gepia.cancer-pku.cn) was utilized to examine the expression levels of the NOTCH1, TOP2A, CD44, PTPRC, CDK4, HNRNPU, and PDGFRA genes in 163 GBM tumor samples and 207 healthy samples obtained from the TCGA (The Cancer Genome Atlas) database. The expression levels of all genes were fully confirmed (Figure 7).

**Figure 8.**
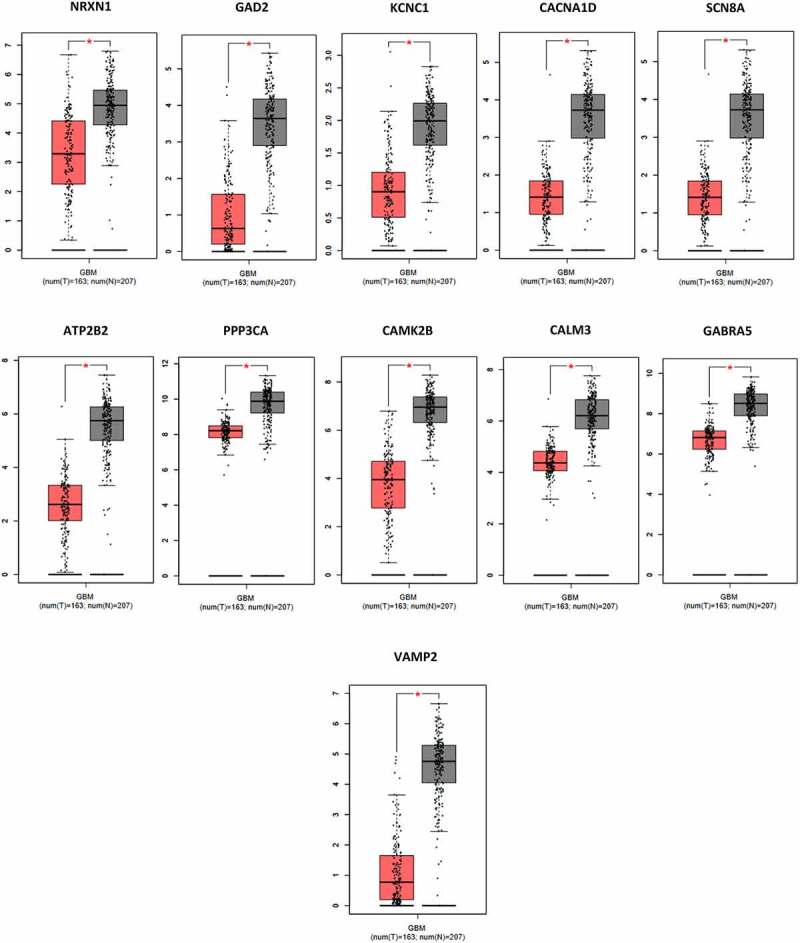
Boxplot of the expression levels of downregulated key and druggable genes (NRXN1, GAD2, KCNC1, CACNA1D, SCN8A, ATP2B2, PPP3CA, CAMK2B, CALM3, GABRA5, and VAMP2) in tumor tissue (red plot) have decreased compared to healthy control samples (gray plot).

Furthermore, the key genes that were found to have decreased expression were analyzed using the GEPIA server. The decrease in expression of these genes was confirmed in the analysis (Figure 8).

**Figure 9.**
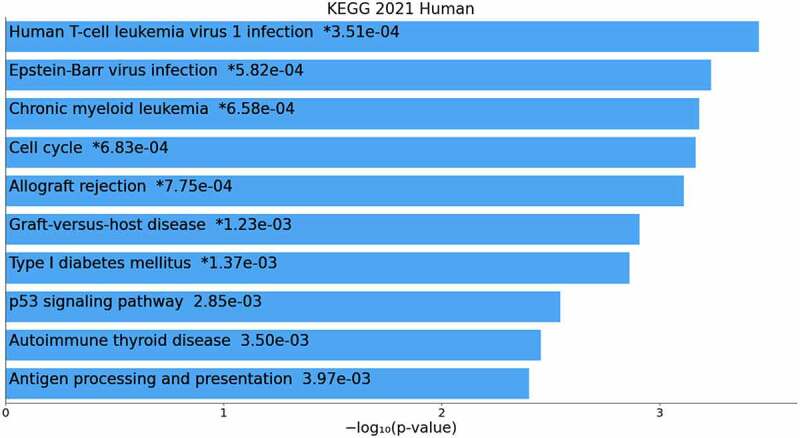
The first ten pathways identified are related to genes involved in expression regulation.

To determine the functional characteristics of the 377 genes that showed increased expression, resulting from the combination of two microarray expression datasets, and to identify the biological processes related to them, we utilized the online Enrichr server for pathway and biological process analysis in GBM, using the KEGG database. Figure 9 displays the top ten pathways and biological processes with a significant P-value <.05, which were identified as the primary targets in GBM.

Additionally, in Table 2, a list of 10 biological processes and their corresponding pathways are mentioned, as well as the genes associated with each pathway.

**Table 2.** List of 10 mentioned biological processes and their corresponding genetic pathways and genes.

Term	P-Value	Q-Value	Overlap genes
Human T-cell leukemia virus 1 infection	.000351	0.036912	[HLA-C,CDC20,IKBKB,CDK4,CHEK2,MYC,CEREB3L2,TSPO,TCF3,TLN1 HLA-DOA,HLA-DPAI,HLA-DQB1]
Epstein-Barr virus infection	.000582	0.036912	[IKBKB,CDK4,MYC,HLA-C,FAS,HES1,CD58,HLA-DOA,CD44,HLA-DPA1,DDB2,HLA-DQB1]
Chronic mye.oid leukemia	.000658	0.036912	[IKBKB,SHC4,MECOM,CDK4,MYC,DDB2,RUNX1]
Cell cycle	.000683	0.036912	[CDC20,CDK4,CHEK2,MYC,SFN,CDC6,SMC3,SMC1A,CDO25B]
Allograft rejection	.000775	0.036912	[HLA-C,FAS,HLA-DOA,HLA-DPA1,HLA-DQB1]
Graft-versuse-host disease	.001234	0.046733	[HLA-C,FAS,HLA-DOA,HLA-DPA1,HLA-DQB1]
Type I diabetes pathway	.001374	0.046733	[HLA-C,FAS,HLA-DOA,HLA-DPA1,HLA-DQB1]
P53 signaling pathway	.002851	0.084822	[CDK4,CHK2,PMAP1,FAS,SFN,DDB2]
Autoimmune thyroid disease	.003502	0.092619	[HLA-C,FAS,HLA-DOA,HLA-DPA1,HLA-DQB1]
Antigen processing and presentation	.003971	0.094499	[CD74,KLRC3,HLA-C,HLA-DOA,HLA-DPA1,HLA-DQB1]

**Figure 10.**
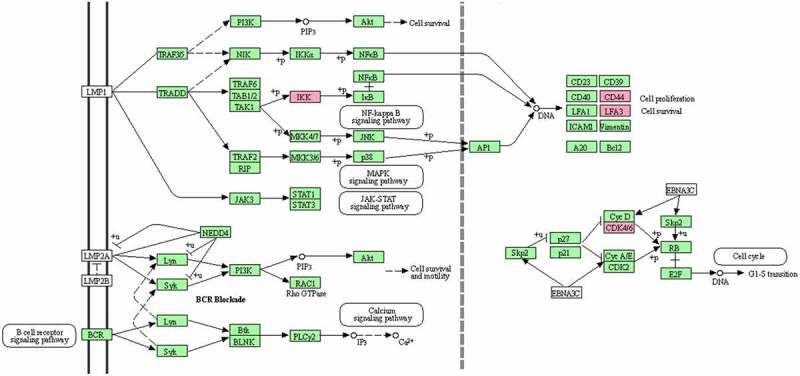
The Epstein-Barr virus biological pathway obtained from the KEGG database. The genes found to be highly expressed in this pathway as a result of the current study, which are likely to be involved in the resistance against GBM, are highlighted in a specific color.

**Figure 11.**
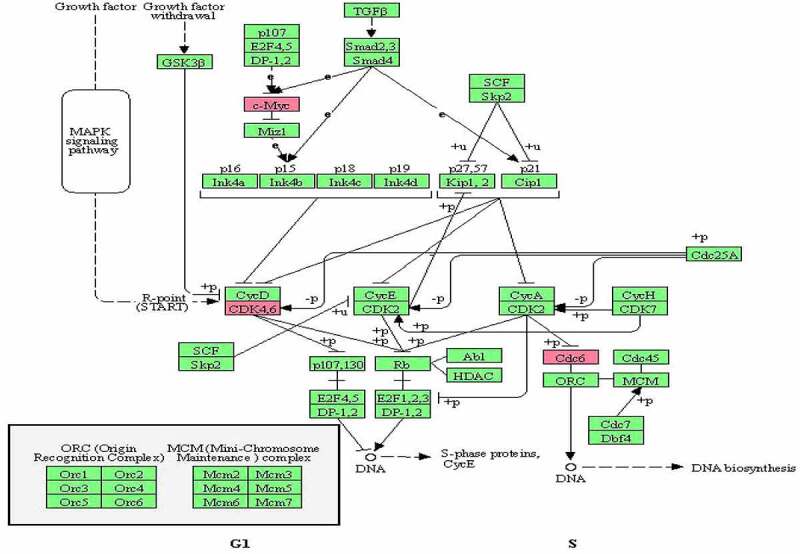
The cell cycle biological pathway obtained from the KEGG database.

**Figure 12.**
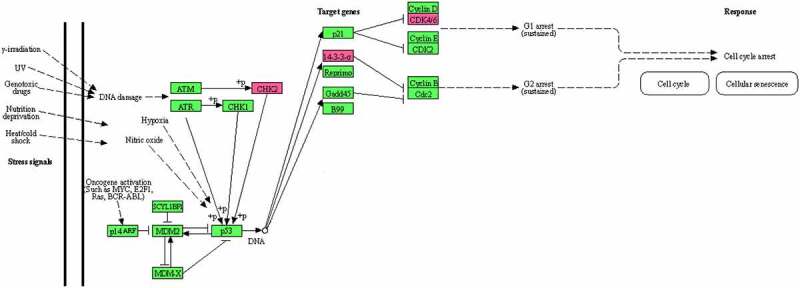
The P53 signaling pathway obtained from the KEGG database.

The Epstein-Barr virus (Figure 10), Cell cycle (Figure 11), and P53 signaling (Figure 12) pathways, which were obtained from the KEGG database, have been shown to be influential on GBM in the following study.

## Discussion

GBM remains a challenging and aggressive brain tumor with limited treatment options, leading to a poor prognosis for patients. The advancements in genomic analysis have provided valuable insights into the underlying mechanisms of GBM, allowing for the identification of potential biomarkers for early diagnosis and treatment. In our study, we analyzed gene expression profiles from two datasets, GSE108474 and GSE50161, comprising GBM and normal samples. This analysis led to the identification of 695 DEGs, including 377 up-regulated and 318 down-regulated genes. The KEGG pathway analysis revealed that the key genes identified in our study are crucial players in the pathogenesis of GBM. Notably, the CD44 gene, which plays a significant role in cell proliferation and survival, was identified as one of the up-regulated genes. Additionally, the KEGG pathway analysis highlighted the involvement of CDK4 in the MAPK signaling pathway, further emphasizing its relevance in tumor growth. The findings from our study suggest that tumor progression in GBM is a complex process, driven by distinct genetic alterations and dysregulation of multiple genes. Rapid cell proliferation is a fundamental aspect of tumor growth, and disruptions in cell cycle regulation can initiate and drive tumor development.^15^

Therefore, understanding the specific molecular mechanisms and genes associated with the cell cycle and P53 signaling pathways may be crucial for future research and targeted therapies in GBM.^16^ Overall, our study contributes to the growing body of knowledge about GBM, providing insights into potential therapeutic targets and pathways that can aid in the development of more effective treatment strategies. However, further research is needed to unravel the intricate molecular mechanisms behind GBM’s aggressiveness and to identify new avenues for therapeutic intervention.

### NOTCH1 protein

The study findings highlight the significance of NOTCH1 as a key gene in the regulatory network associated with the prognosis of GBM patients. The expression of NOTCH1 is found to be higher in patients who have survived for more than one year compared to those with a survival period of less than one year. The NOTCH1 family plays a crucial role in controlling cell fate decisions during various growth processes. NOTCH1 is a part of the NOTCH signaling network, an intercellular signaling pathway conserved to regulate interactions between neighboring cells.^17^ During development, activation of NOTCH1 before birth leads to the differentiation of radial glia, while after birth, it leads to the differentiation of astrocytes.^18^ Additionally, NOTCH1 is found to be more highly expressed in glioblastoma stem cells (GSCs) located in the tumor periphery as opposed to those in the tumor core.^19,20^

The observed higher expression of NOTCH1 in GBM patients with a longer survival duration may suggest its potential role as a prognostic biomarker in GBM. The differential expression of NOTCH1 in different regions of the tumor and its association with GSCs could provide valuable insights into the tumor’s heterogeneity and stem cell population. Targeting NOTCH1 signaling pathways could offer new therapeutic opportunities in GBM management. However, further research is required to elucidate the specific mechanisms by which NOTCH1 contributes to GBM prognosis and to explore its potential as a therapeutic target. Understanding the intricate regulatory functions of NOTCH1 in GBM could lead to the development of novel treatment strategies aimed at improving patient outcomes and survival rates.

### MYC protein

The MYC protein has been identified as another hub in the study. The MYC gene family, consisting of MYCN, MYC, and MYCL, comprises strong oncogenes that play a critical role in the pathogenesis of various human cancers.^21^ In both normal cells and cancer cells, the MYC transcription factor collaborates with the nucleophosmin (NPM) protein to regulate cell growth and division. The formation of a complex called MYC-NPM allows MYC to directly interact with the target MYC promoter genes, leading to the transcription of essential proteins for cellular transformation.^22^ The function of MYC in gene regulation is remarkable. Genes that are expressed at moderate levels in the absence of MYC are significantly upregulated in the presence of MYC, while genes expressed at low levels in the absence of MYC show only minor enhancement in its presence.^23^ In GBM, MYC plays a crucial role in enhancing the self-renewal capability of neurosurgeons and maintaining the tumorigenicity of these cells. A subgroup of GBM is characterized by high expression levels of MYC and MYCN genes, indicating their potential as therapeutic targets in GBM.^24^ The identification of MYC as a hub in the protein-protein interaction network suggests its central role in GBM pathogenesis. Targeting MYC signaling pathways may hold promise as a therapeutic strategy for GBM treatment. However, the complexity of MYC functions in various cellular processes requires further investigation to fully comprehend its involvement in GBM development and progression. Advancements in understanding the regulatory mechanisms of MYC could open up new avenues for developing targeted therapies to improve the prognosis and management of GBM patients.

### TOP2A protein

TOP2A is a nuclear enzyme that plays a crucial role in various cellular processes, including chromosomal condensation, chromatid separation, and the relaxation of stress and strain during DNA replication and repair.^25^ As a sensitive and specific marker of actively dividing cells, TOP2A is predominantly active during the final stages of the cell cycle, including G2, S, and M phases. This protein has been implicated in a wide range of human cancers, including gliomas. Studies using data from the Oncomine database have shown that TOP2A is significantly overexpressed in glioma tissue compared to normal controls. This overexpression suggests that TOP2A may play a critical role in the development and progression of gliomas. Moreover, the regulatory role of TOP2A in the expression of other genes further supports its importance as a potential predictive or therapeutic target in gliomas.^26^ Given its involvement in fundamental cellular processes and its association with glioma pathogenesis, TOP2A emerges as an attractive target for the development of novel therapeutic approaches for glioma treatment. By understanding the molecular mechanisms underlying the dysregulation of TOP2A in gliomas, researchers may identify potential strategies to modulate its activity or expression, leading to improved therapeutic outcomes for patients with this aggressive brain tumor. Further investigations into the functional role of TOP2A and its interaction with other key genes identified in this study could provide valuable insights into the complexity of GBM pathogenesis and aid in the development of targeted therapies for effective GBM management.

### CD44 protein

CD44 is a multifunctional glycoprotein that plays a pivotal role in various cellular processes, including cell movement, proliferation, apoptosis, and angiogenesis. Its involvement in these processes makes it a crucial regulator of cell behavior and tissue homeostasis. However, in the context of oncogenic MYC, CD44 becomes a poor prognostic marker. Notably, CD44 is widely expressed in many types of cancer, including skin, blood, head and neck, lung, breast, stomach, large intestine, prostate, uterus, and brain.^27^ Its diverse expression across various cancer types underscores its significance in cancer pathogenesis and progression. In glioblastoma (GBM), CD44 s extracellular domain directly interacts with hyaluronic acid (HA) and other matrix factors, facilitating the migration of GBM cells.^28^ Moreover, CD44 can also interact with other ligands, such as osteopontin, collagen, and matrix metalloproteinases (MMPs).^29^ These interactions contribute to the invasive and metastatic properties of GBM cells, making CD44 a key player in the aggressive behavior of this deadly brain tumor. Given its widespread expression in cancer and its essential role in promoting cell migration and invasion in GBM, CD44 holds promise as a potential therapeutic target. Targeting CD44 could offer new avenues for the development of innovative anti-cancer therapies aimed at disrupting tumor progression and metastasis. Further investigations into the specific mechanisms by which CD44 interacts with its ligands and modulates cellular processes in GBM may unveil novel therapeutic strategies for managing this devastating disease. Moreover, understanding the interplay between CD44 and other identified key genes in GBM could provide valuable insights into the intricate molecular pathways governing GBM pathogenesis and guide the development of precision therapies tailored to individual patients needs.

### PTPRC protein

The PTPRC gene encodes for the protein tyrosine phosphatase C (PTPRC), commonly known as CD45. CD45 is a crucial regulator of T cell activation through its anti-gene receptor activity.^30^ As a member of the Tyrosine Phosphatase (PTP) family, CD45 is a signaling molecule that plays a significant role in regulating various cellular processes, including cell growth, differentiation, mitosis, and metabolic conversion.^31^ In the human genome, there are 107 genes belonging to the PTP enzyme family, and 15 of these genes have been found to be significantly involved in the development of glioma, a type of brain tumor.^32^ The ample evidence of the involvement of PTP enzymes, including CD45, in the development and progression of tumors, including gliomas, underscores their potential as important therapeutic targets for high-grade glioma treatment.^33,34^ By interfering with the biology of PTP enzymes, researchers may discover novel strategies for the detection and treatment of gliomas. Understanding the precise role of CD45 and other PTP enzymes in glioma development could lead to the development of targeted therapies that specifically address the underlying molecular pathways responsible for tumor growth and invasion. Moreover, further exploration of the interactions between CD45 and other identified key genes in glioma may shed light on the complex network of signaling pathways driving tumor progression. Such insights may pave the way for personalized medicine approaches that tailor treatment strategies to individual patients based on their unique genetic profiles. As we delve deeper into the molecular mechanisms governing glioma pathogenesis, the potential for innovative and effective therapeutic interventions becomes increasingly promising.

### SSRP1 protein

Studies have consistently demonstrated that SSRP1, both at the mRNA and protein levels, is significantly elevated in glioma tissue compared to normal brain tissue. Moreover, the levels of SSRP1 are notably higher in patients with high-grade glioma compared to those with low-grade glioma.^35^ These findings indicate that SSRP1 may play a critical role in the progression of GBM. To further elucidate the involvement of SSRP1 in the advancement of GBM cells, various techniques, such as CCK-8 and EdU assays, as well as FACS analysis, have been employed.^36^ The results have revealed that silencing SSRP1 through siRNA leads to a significant reduction in the survival and replication capabilities of glioma cells. Additionally, inhibiting SSRP1 has been shown to suppress the migration and invasion of glioma cells.^37^ These observations suggest that SSRP1 may contribute to the aggressive behavior of GBM cells, making it a potential therapeutic target for combating the disease.

### CDK4 protein

The CDK4-cyclinD and CDK6-cyclinD complexes are pivotal in phosphorylating and inactivating the tumor suppressor protein retinoblastoma (RB). This inactivation of RB diminishes dependence on E2F and promotes the expression of genes essential for the transition from G1 to S phase of the cell cycle. Consequently, CDK4 and CDK6 have become prime targets for therapeutic interventions in cancer treatment. Notably, changes in the CDKN2A/CCND2/CDK4/CDK6 pathway are frequently observed in GBM. Alterations in this pathway can significantly influence cell cycle regulation and contribute to the uncontrolled proliferation of cancer cells in GBM.^38^ Understanding the role of CDK4 in the context of GBM pathogenesis may lead to the development of targeted therapies aimed at inhibiting its activity. By targeting key components of the CDK4 pathway, it may be possible to halt the cell cycle progression of GBM cells and suppress tumor growth. Such targeted therapies offer promising potential in the treatment of GBM, as they focus on disrupting the specific molecular pathways that drive tumor development and progression.

### DKC1 protein

The DKC1 protein belongs to the H/ACA snoRNPs family, which plays a crucial role in the processing and modification of rRNA. snoRNPs are classified into two families: C/D and H/ACA.^39^ The DKC1 gene, located at Xq28 on the X chromosome, is responsible for the formation of specific small RNAs and telomerase activity.^40^ Inherited mutations in the DKC1 gene result in the inactivation of the protein and lead to dyskeratosis congenita, a congenital skin disorder characterized by skin abnormalities, blood cell dysfunction, and increased susceptibility to certain forms of cancer.^41^ Interestingly, the regulatory functions of the DKC1 gene have been implicated in various forms of human cancer, including ovarian and breast cancer.^42^ The study findings reveal a significant increase in the expression of the DKC1 gene in pathological glioma tissue compared to normal tissue. This suggests that DKC1 may play a vital role in glioma development and progression. Further investigations into the specific mechanisms by which DKC1 influences glioma pathogenesis could shed light on potential therapeutic targets for glioma treatment.

### HNRNPU protein

The HNRNPU protein is a prevalent nucleoplasmic protein and represents the largest primary protein of hnRNP (heterogeneous nuclear ribonucleoprotein). HNRNPU is known to bind to pre-mRNA within the cell and can bind to both RNA and single-stranded DNA in laboratory conditions.^43^ Substantial evidence indicates that hnRNP A2/B1, a member of the hnRNP family, plays a crucial role in the development and progression of various human cancers. The data from this study reveal that hnRNP A2/B1 is overexpressed in glioma tissue samples and is associated with advanced stages of glioma.^44^ Moreover, inhibiting hnRNP A2/B1 could potentially reduce the survival, adhesion, migration, invasion, and chemoresistance of GBM cells, particularly in response to TMZ treatment.^45^ These findings suggest that targeting hnRNP A2/B1 may offer a promising strategy for mitigating GBM progression and enhancing the efficacy of chemotherapy. By understanding the involvement of DKC1 and HNRNPU in the context of glioma biology, researchers may gain valuable insights into the underlying mechanisms of glioma pathogenesis. Additionally, these proteins may serve as potential targets for the development of novel therapeutic approaches for combating GBM.

### PDGFRA protein

The PDGFRA gene is known to be associated with somatic mutations that result in its fusion with specific genes in blood-forming stem cells, leading to blood disorders in the category of chronic myeloproliferative neoplasms.^46^ Notably, most proneural GBMs harbor a mutation in TP53, in conjunction with overexpression of PDGFRA.^47^

In a study conducted by Yang Zhang and colleagues in 2017, crucial oncogenes linked to the progression of GBM were identified, and their mechanisms were established. The data on gene expression from GSE50161, obtained from the GEO database, were examined by the researchers. A total of 486 genes related to cancer and genetic performance in GBM were identified, with 128 genes showing positive regulation and 358 genes showing negative regulation. Gene Ontology (GO) and KEGG analysis were utilized to determine the functions and enrichment of these genes. The analysis revealed that the positively regulated differentially expressed genes (DEGs) were associated with processes such as cellular division, cellular proliferation, intermediate filaments, nucleoplasm, protein binding, and protein kinase activity. Additionally, the key KEGG pathways indicated that the upregulated DEGs participated in cancer-related apoptotic pathways, complementation and cascades, as well as the p53 signaling pathway and cell cycle. The regulated DEGs, as per the GO and KEGG analysis, may be linked to the advancement and growth of GBM. Specifically, genes such as CDK1, CCNB1, and CDC20 have shown increased expression and are significantly associated with a negative prognosis in patients.^48,49^ CDK1 reduction was discovered by Yang et al. to have a significant effect on the suppression of GBM proliferation, and the study suggested that CDK1 is involved in the Akt signaling pathway.^16,50^ Additional research is warranted to further understand the identification and management of GBM, and the mechanisms underlying GBM are providing valuable insights.^51^

Further research conducted by Kai Cui et al. focused on using bioinformatic technology to identify potential genetic targets for the diagnosis and treatment of GBM. The study data was obtained from patients with GBM, while the control data was acquired from the GEO database. Through GEPIA and GEO2R analysis, the researchers identified 130 DEGs and 10 hub genes. The analysis revealed significant correlations among CCNB1, CDC6, KIF23, and KIF20A, indicating strong connections between them.^52^ Importantly, in patients with GBM, all four genes were expressed at markedly higher levels than in the control group, as demonstrated by RT-qPCR. According to Cui et al., CCNB1, CDC6, KIF23, and KIF20A genes can serve as effective biomarkers for the identification and management of GBM.^52^

## Conclusion

Our study has provided valuable insights into the genetic mechanisms underlying GBM and has identified several key genes and pathways associated with the disease. The analysis of differentially expressed genes (DEGs) revealed the importance of pathways such as cell cycle and P53 signaling in GBM pathogenesis. Among the key genes identified, MYC, NOTCH1, TOP2A, CD44, PTPRC, SSRP1, CDK4, DKC1, HNRNPU, and PDGFRA have shown significant correlations with GBM prognosis and may serve as potential targets for therapeutic interventions. The identification of these key genes and pathways opens new avenues for the development of targeted treatments for GBM. However, further experimental studies are necessary to validate their potential as therapeutic targets and to understand the specific molecular mechanisms involved in GBM development and progression.

In summary, our bioinformatic analysis provides a foundation for future research and clinical investigations aiming to improve the diagnosis and treatment of GBM. The complex network of biological pathways involved in GBM requires continued exploration to unlock its full potential for effective therapeutic strategies and improved patient outcomes.
